# Analytical validation of a metagenomic next-generation diagnostic platform for urinary tract infection in a Thai tertiary hospital setting: a BI-Biotia UTI cohort study

**DOI:** 10.3389/fcimb.2026.1751074

**Published:** 2026-02-13

**Authors:** Panupong Wangprapa, Dorottya Nagy-Szakal, Heather L. Wells, Gabor Fidler, Montinee Sangtian, Wipa Panmontha, Srichan Bunlungsup, Wichai Techasathit, Mara Couto-Rodriguez, David C. Danko, Christopher E. Mason, Niamh B. O’Hara, Sira Sriswasdi, Teeradache Viangteeravat

**Affiliations:** 1 Bumrungrad International Hospital, Bangkok, Thailand; 2 Inter-Department Program of Biomedical Sciences, Faculty of Graduate School, Chulalongkorn University, Bangkok, Thailand; 3 Center of Excellence in Computational Molecular Biology, Faculty of Medicine, Chulalongkorn University, Bangkok, Thailand; 4 Genomic Medicine Center, Bumrungrad International Hospital, Bangkok, Thailand; 5 Biotia Inc., New York, NY, United States; 6 The Department of Cell Biology, College of Medicine, SUNY Downstate Health Sciences University, New York, NY, United States; 7 VitalLife Scientific Wellness Center, Bangkok, Thailand; 8 Center for Artificial Intelligence in Medicine, Research Affairs, Faculty of Medicine, Chulalongkorn University, Bangkok, Thailand

**Keywords:** antibiotic stewardship, antimicrobial resistance, high risk UTI patients, long read sequencing, metagenomic sequencing, Oxford Nanopore Technology, urinary tract infection

## Abstract

**Background:**

The BIOTIA-DX platform (BDX), a commercially available clinical-grade mNGS-based test in the United States, has not been analytically validated for urinary tract infections (UTIs) in a Southeast Asian cohort, where microbial epidemiology and antimicrobial resistance (AMR) patterns differ significantly.

**Objective:**

Our primary objective was to evaluate the analytical performance and concordance with standard urine culture of the BIOTIA-DX platform in a Thai tertiary hospital setting, thereby assessing its transportability to a Southeast Asian population with distinct microbial epidemiology.

**Methods:**

We analyzed 398 retrospectively collected urine samples from patients with suspected UTI at a private hospital in Bangkok. Each sample was processed in parallel using standard-of-care urine culture and the BDX mNGS workflow. After excluding 30 samples with insufficient sequencing reads (<500 non-human reads), 368 samples (231 culture-positive, 137 culture-negative) were included. Diagnostic accuracy was assessed against culture, and genotypic AMR predictions were compared to phenotypic antimicrobial susceptibility testing (AST) N = 192.

**Results:**

The BIOTIA-DX platform demonstrated high analytical sensitivity at the sample level (98.7% [95% CI: 0.95-0.99]; 228/231 culture-positive samples detected) and organism level (94.6%; 229/242 culture-identified organisms correctly detected). Among 137 culture-negative samples, BIOTIA-DX detected microbial DNA in 98 samples (71.5%), identifying 264 organisms not detected by standard culture. These additional detections predominantly comprised anaerobic organisms (150/264, 56.8%) and fastidious species (54/264, 20.5%); however, the clinical significance of these detections (infection vs. colonization vs. contamination) could not be determined without clinical correlation. For AMR prediction, genotype-phenotype concordance rates were 94.1% for fluoroquinolone resistance in *E. coli* (96/102 resistant isolates correctly predicted), 91.4% for beta-lactams (106/116), 91.3% for aminoglycosides (21/23), and 81.5% for sulfamethoxazole/trimethoprim (75/92). Specificity and positive predictive value could not be calculated because organisms detected by BIOTIA-DX but not by culture could not be definitively classified as true positives or false positives without independent confirmation.

**Conclusions:**

The BIOTIA-DX platform demonstrates robust analytical concordance with urine culture in a Thai patient population. Prospective clinical validation studies are needed to assess clinical utility and impact on patient outcomes, particularly in culture-negative and polymicrobial cases. This study represents the first analytical validation of this platform using Oxford Nanopore Technology and the first validation in Southeast Asia.

## 1 Introduction

Traditional urine culture (UC) methods are often too slow to guide antibiotic therapy in patients with Urinary Tract Infection (UTI), which affects two-thirds of the female population at some point in their lives (Bauer et al., 2014; Medina and Castillo-Pino, 2019). Additionally, conventional methods often fail to detect fastidious or uncultivable pathogens and may miss polymicrobial infections, wherein multiple organisms contribute to disease. These diagnostic limitations can lead to empirical treatment failure and further exacerbate the global challenge of antimicrobial resistance, emphasizing the need for a paradigm shift in infectious disease diagnostics and management (Bauer et al., 2014; Brubaker and Wolfe, 2015; Sathiananthamoorthy et al., 2019). Metagenomic next-generation sequencing (mNGS) has emerged as a transformative, culture-independent approach, which has some advantages when compared to selective culture and other molecular methods like PCR. Long read sequencing is also capable of providing rapid, comprehensive pathogen identification and phased AMR gene profiling within the detected organism.

In the United States, commercial mNGS platforms like BIOTIA-DX (Biotia, Inc., NY) are using mNGS to address microbial diagnostic challenges. These platforms often leverage proprietary bioinformatics pipelines and machine learning models trained on data from specific patient populations to enhance accuracy (Romero et al., 2023; Couto-Rodriguez et al., 2025; Fidler et al., 2025). However, a critical unanswered question is whether these sophisticated diagnostic systems, developed and validated in one geographical region, are directly transportable to international settings with distinct host genetics, environments, and microbial landscapes. The microbial epidemiology and AMR patterns in Southeast Asia, for example, differ significantly from those in North America.

Specifically, southeast Asia (SEA), with a population of around 500 million, has been reported to be at greater risk of AMR (Sihombing et al., 2023) and resistance patterns differ drastically. For example, ciprofloxacin resistance in SEA ranges from 50-90% (Sugianli et al., 2021), compared with 8-20% in the United States (Ku et al., 2024), and higher concentrations of ESBL have also been reported (Chen et al., 2019).

In Thailand, the high prevalence of resistance to first-line antibiotics, such as fluoroquinolone resistance in *E. coli* exceeding 40%, renders many international treatment guidelines inapplicable and fuels the demand for advanced, localized diagnostic strategies (Sangsuwan et al., 2021; Anugulruengkitt et al., 2023; Assawatheptawee et al., 2022). Due to differences in microbial epidemiology, host genetics, and antimicrobial resistance patterns, machine learning models trained primarily on US patient cohorts may not perform optimally in Southeast Asia populations without rigorous local validation.

Our primary objective was to conduct an analytical validation of the BIOTIA-DX platform against standard urine culture in a real-world setting at a private tertiary hospital in Bangkok, Thailand. By comparing its performance directly against the standard of care, we aimed to assess the platform’s analytical sensitivity, concordance with culture-based methods, and overall transportability to a new and diverse international patient population with different microbial epidemiology and AMR patterns, providing essential evidence for its potential global deployment.

This analytical validation study establishes baseline performance characteristics necessary before prospective clinical validation studies can be conducted to assess clinical utility, treatment outcomes, and impact on antimicrobial stewardship.

## 2 Methods

### 2.1 Study design and participants

A retrospective cohort dataset was obtained from 398 patients who visited a tertiary care private hospital in Bangkok for suspected UTI or routine monitoring between January 2018 and December 2020. The characteristics of the cohort are presented in **Table 1**; **Supplementary Table S1**. The median age of the overall cohort was 64 years, and the majority of patients were female (277, 69.6%). Samples were included for analysis if they contained more than 500 non-human reads after preprocessing and removal of human DNA sequences. A total of 368 samples satisfied these criteria and were included in the final analysis. The Bumrungrad International Hospital’s Institutional Review Board reviewed and approved the study, which used anonymized data (BI-IRB #312-02–23 CIEN-B Fub). No formal power calculation was performed for this retrospective analytical validation. Sample size (N = 398) was determined by available archived samples meeting inclusion criteria during the study period.

**Table 1:** Cohort characteristic (N = 398).

Characteristic	Overall cohort (N = 398)	Geographic region	Overall cohort (N = 398)
Age	years		Geographic Region, n (%)
Mean (SD)	62.5 (18.4)	Thailand	161 (40.5%)
Median (IQR)	64.0 (49.2–76.0)	Southeast Asia (non-Thai)	68 (17.1%)
Age Group	n (%)	Middle East	69 (17.3%)
<40 years	56 (14.1%)	South Asia	26 (6.5%)
40–59 years	108 (27.2%)	Europe	28 (7.0%)
60–79 years	155 (38.9%)	East Asia	17 (4.3%)
≥80 years	79 (19.8%)	North America	15 (3.8%)
Sex	n (%)	Oceania	4(1.0%)
Female	277 (69.6%)	Africa	5 (1.3%)
Male	121 (30.4%)	others	3 (0.8%)

### 2.2 Standard of care diagnostics (urine culture and AST)

Urine was streaked onto blood agar and MacConkey agar without centrifugation. Plates were incubated aerobically at 35 °C for 24 hours. Pathogen identification used MALDI-TOF MS (VITEK MS v3.2, bioMérieux). We utilized a threshold of ≥10^3^ CFU/mL to define a positive urine culture. This threshold was selected to maximize sensitivity for the detection of potential uropathogens. Antimicrobial susceptibility testing employed VITEK 2 system (bioMérieux) with turbidimetric monitoring for up to 36 hours to determine minimum inhibitory concentrations.

### 2.3 Nanopore metagenomic sequencing

Ten-milliliter urine aliquots were processed within 4 hours. Human cells were removed by centrifuge (300g×2min), then microbes pelleted (5000g×15min). Human DNA was depleted using Molysis Basic 5 Kit (Molzym D-301-100); microbial DNA was extracted using Maxwell RSC Viral kit (Promega AS1330). Following extraction, DNA was stored at -20 °C to facilitate sample batching. Samples ≥ 0.1ng/µL underwent library preparation using ONT Rapid PCR Barcoding Kit (SQK-RPB004, 14 cycles). Pooled libraries were sequenced on MinION flow cells (FLO-MIN106D R9.4.1) using MinKNOW v22.12 for 24h, with 6 multiplexed. All urine specimens were processed internally at the CAP-accredited Bumrungrad International Hospital laboratory by well-trained laboratory personnel, ensuring consistency and reliability in sample handling and analytical workflow (https://www.bumrungrad.com/laboratory/en).

The 500 non-human read threshold was established to ensure sufficient sequencing depth for reliable organism identification by the BIOTIA-DX classifier. There were 30 samples that failed this threshold, 8 of which were due to low DNA yields (<0.5 nq/uL). The other 22 samples exhibited normal DNA yields but may have failed at library preparation or sequencing stages.

### 2.4 BIOTIA-DX, a clinical-grade metagenomic tool

After preprocessing steps removing low-quality reads and reads mapping to the human genome, the BIOTIA-DX (BDX) pipeline identifies microbes present in a metagenomics sample through a two-step process. First, the non-human reads are aligned to a large database of microbial genomes in a coarse classification step. Organisms passing a pre-determined threshold in coarse classification are then used in a fine classification step which uses machine learning (Couto-Rodriguez et al., 2022, 2024) to statistically quantify whether the organism is present or absent in the sample. Reads are aligned to curated pangenomes (Hyun et al., 2022) for each organism identified in the coarse classification step and summary statistics describing alignment quality and genome coverage are calculated for each organism. These statistics are then fed into the machine learning classifier which assigns a confidence score ranging from 0 to 1 for whether the organism was present (score=1) or absent (score=0). The BIOTIA-DX machine learning classifier was intentionally trained and designed to increase stringency and decrease false positive detection of urogenital commensals or opportunistic pathogens present at colonization levels. Organisms with a BIOTIA-DX confidence score greater than 0.5 were considered to be positive identifications. In addition, the pipeline is designed to provide identification as soon as sufficient reads are generated.

### 2.5 BIOTIA-DX antimicrobial resistance gene detection

The BDX pipeline covers general detection of some of the most prevalent and relevant gene markers in urine, including beta-lactamases (blaOXA, blaNDM, blaCTX-M, blaTEM, blaKPC, blaCMY, blaADC, blaSHV, blaVIM, blaPDC, blaZ, cfxA), *mec* gene classes (mecA-D), aminoglycoside, sulfonamide (sul1-4) and trimethoprim (dfr) resistance markers. A comprehensive database was constructed from the AMRFinder and CARD databases (Feldgarden et al., 2021; Alcock et al., 2022). As a first step, sequencing reads are aligned to AMR gene clusters, and alignment statistics are extracted. After filtering alignments with low-quality metrics, gene sequences are reconstructed and translated into amino acid sequences. Tripeptide fragments of the protein sequences are then fed into a shallow neural network for gene identification. Additionally, fluoroquinolone resistance in *E. coli* is monitored using a protein variant-based machine learning classifier trained on isolates from the BV-BRC database (Couto-Rodriguez et al., 2024). The model encompasses mutations in *gyrA*, *gyrB*, *parC*, and *parE* proteins.

### 2.6 Evaluation of BIOTIA-DX against urine culture and AST

For comparison of organisms identified by culture and by BIOTIA-DX, we considered cases where culture and BIOTIA-DX identified the same organism as a true positive. Where culture and BIOTIA-DX were both negative, we considered the case a true negative. Where culture identified an organism that was not identified by BIOTIA-DX with prediction probability > 0.5, we labeled the case false negative. In cases where a culture-identified organism did not pass the coarse classification stage of BIOTIA-DX and thus was not assigned a prediction probability during the fine classification stage, we excluded the organism from the analysis. As we cannot definitively determine whether organisms identified by BIOTIA-DX but not by culture are false positive identifications by BIOTIA-DX or false negative detections by culture, we simply counted these organisms as ‘additional positives.’ Thus, we were able to calculate the sensitivity of BIOTIA-DX compared to culture (true positives/true positives + false negatives), but we were not able to calculate a value for specificity.

We also assessed the performance of BIOTIA-DX at the overall sample level. If at least one microbe was identified by culture and at least one microbe was detected by BIOTIA-DX, we considered the sample to be in agreement between the two methods. The sample was considered to be a complete agreement if all organisms detected by culture were also detected by BIOTIA-DX. In the case that some, but not all microbes identified by culture were identified by BIOTIA-DX (or vice versa), the sample was considered in partial agreement. This classification was applied when both platforms identified members of the same established taxonomic complex or genus such as the *Acinetobacter baumannii* (ACB) or *Klebsiella pneumoniae* (KpSC) complexes where high proteomic similarity often limits the discriminative power of MALDI-TOF MS. Thus, partial agreement reflects concordance at the genus/complex level while acknowledging the superior species-level resolution of mNGS. The sample was also considered a partial agreement if the two methods both found the sample to be positive but identified the species differently. Finally, the methods were considered in disagreement when culture identified one or more microbes, but BIOTIA-DX did not identify any.

For comparison of AMR calls, we considered BIOTIA-DX and AST to be in agreement if either of the following were true: the isolate identified by culture was marked as intermediate or resistant to at least one tested drug in a drug class and BIOTIA-DX identified at least one antimicrobial resistance gene (ARG) to that drug class; OR the isolate identified by culture was susceptible to all tested drugs in a drug class and BIOTIA-DX did not identify any ARGs to that drug class. In any case where BIOTIA-DX did not identify resistance genes in a phenotypically resistant or intermediate isolate, we marked the sample as a disagreement. When BIOTIA-DX identified ARGs in a sample where the isolate was susceptible by AST, we also marked the sample as a disagreement; however, many of these samples contained multiple organisms, so we noted that it was possible that the discrepant ARGs belonged to a different organism in the same sample. For isolates found to be resistant to drugs that were only tested in combination (e.g., sulfamethoxazole/trimethoprim), we required ARGs from both drug classes to be present to mark the sample as an agreement, thereby minimizing the potential for over-interpretation of the genotypic data.

### 2.7 Sample classification criteria

Samples were classified as culture-positive or culture-negative. Culture-positive means any bacterial organism identified by standard culture methods, whereas culture-negative means no bacterial growth on standard urine culture. Fungal organisms were excluded from analysis because the DNA extraction method used (Molysis Basic 5 Kit) does not efficiently lyse fungal cells, making BIOTIA-DX unable to detect fungi regardless of their presence. Additionally, *Corynebacterium imitans* and *Lactobacillus* spp. were excluded as they likely represent urogenital commensals rather than infection. Culture identifications lacking any genomic signal in BIOTIA-DX (no reads aligned in coarse classification, n=24) were excluded from organism-level concordance calculations as likely culture contaminations, while maintaining conservative sample-level classifications.

## 3 Results

### 3.1 Urine culture results

Overall, 24 different bacterial species and 4 fungal species were identified through standard urine culture in this cohort (**Table 2**). *Escherichia coli* was the dominant species (n=157), followed by *Klebsiella pneumoniae* (n=32) and *Enterococcus faecalis* (n=30), all of which are well-known uropathogens. *Staphylococcus* species were the next most common organism detected by culture, identified as *Staphylococcus aureus* (n=5), *Staphylococcus epidermidis* (n=3), *Staphylococcus haemolyticus* (n=2), and *Staphylococcus saprophyticus* (n=1). Other common organisms included *Pseudomonas aeruginosa* (n=10), *Streptococcus agalactiae* (n=6), *Proteus mirabilis* (n=5), *Enterobacter cloacae* (n=5), *Citrobacter koseri* (n=5), *Enterococcus faecium* (n=4), and *Acinetobacter baumannii* (n=4). The remaining 11 bacterial species identified by culture were each detected two or fewer times: *Actinomyces urogenitalis*, *Aeromonas caviae*, *Burkholderia cepacia*, *Citrobacter freundii*, *Corynebacterium imitans*, *Enterococcus raffinosus*, *Klebsiella aerogenes*, *Lactobacillus* species, *Morganella morganii*, *Serratia marcescens*, and *Stenotrophomonas maltophilia*. Fungal species were also common, with *Candida albicans* (n=7) being the most prevalent, followed by *Candida glabrata* (n=3), *Candida tropicalis* (n=2), and *Candida krusei* (n=1).

**Table 2:** Most common organisms identified by urine culture.

Organism type	Organism	Total positive by culture	% of culture-positive	% of all samples
Gram-negative bacteria		218	94.40%	59.24%
	*Escherichia coli*	157	68.00%	42.70%
	*Klebsiella pneumoniae*	32	13.90%	8.70%
	*Pseudomonas aeruginosa*	10	4.30%	2.70%
	*Proteus mirabilis*	5	2.20%	1.40%
	*Enterobacter cloacae*	5	2.20%	1.40%
	*Citrobacter koseri*	5	2.20%	1.40%
	*Acinetobacter baumannii*	4	1.70%	1.10%
Gram-positive bacteria		51	22.10%	13.90%
	*Enterococcus faecalis*	30	13.00%	8.20%
	*Staphylococcus aureus*	5	2.20%	1.40%
	*Streptococcus agalactiae*	6	2.60%	1.60%
	*Enterococcus faecium*	4	1.70%	1.10%
	*Staphylococcus epidermidis*	3	1.30%	0.80%
	*Staphylococcus haemolyticus*	2	0.90%	0.50%
	*Staphylococcus saprophyticus*	1	0.40%	0.30%
Fungi		13	5.60%	3.50%
	*Candida albicans*	7	3.00%	1.90%
	*Candida glabrata*	3	1.30%	0.80%
	*Candida tropicalis*	2	0.90%	0.50%
	*Candida krusei*	1	0.40%	0.30%

### 3.2 Diagnostic performance of BIOTIA-DX compared to urine culture

Samples were considered suitable for BIOTIA-DX analysis if greater than 500 reads were present in the sequencing data after preprocessing and human DNA removal. Of the 398 samples in this study, 30 samples either did not exceed this threshold, were contaminated, or were not sequenced and were excluded, resulting in 368 samples suitable for analysis. Of these 368 samples, 260 were positive by culture and 108 were negative or inconclusive. There were 24 organisms across the culture positive samples where BIOTIA-DX did not find any genomic signal of the organism in the coarse classification stage (**Supplementary Table S2A**). Because lack of a genomic signal is extremely unlikely in samples where an organism is present at the clinical threshold (≥10^3^ CFU/mL), we categorized these 24 cases as culture-positive with no detected genomic signal (a present organism missed by BIOTIA-DX will almost always pass coarse classification but have a prediction probability < 0.5), these identifications may represent false positive identifications by culture and were removed from the analysis. These discrepancies where a culture-positive organism exhibited no detected genomic signal may be partly due to DNA degradation or other preprocessing factors, reflecting the complexities of real-world clinical settings (El Bali et al., 2014; Bundgaard-Nielsen et al., 2020). In 15 of 24 cases, the removal of the culture-positive organism with no detected genomic signal left no culture-positive organisms in the sample, and these samples were reclassified as culture-negative (for bacteria). The remaining 9 cases had at least one additional organism called by culture and remained classified as culture positive.

**Figure 1:**
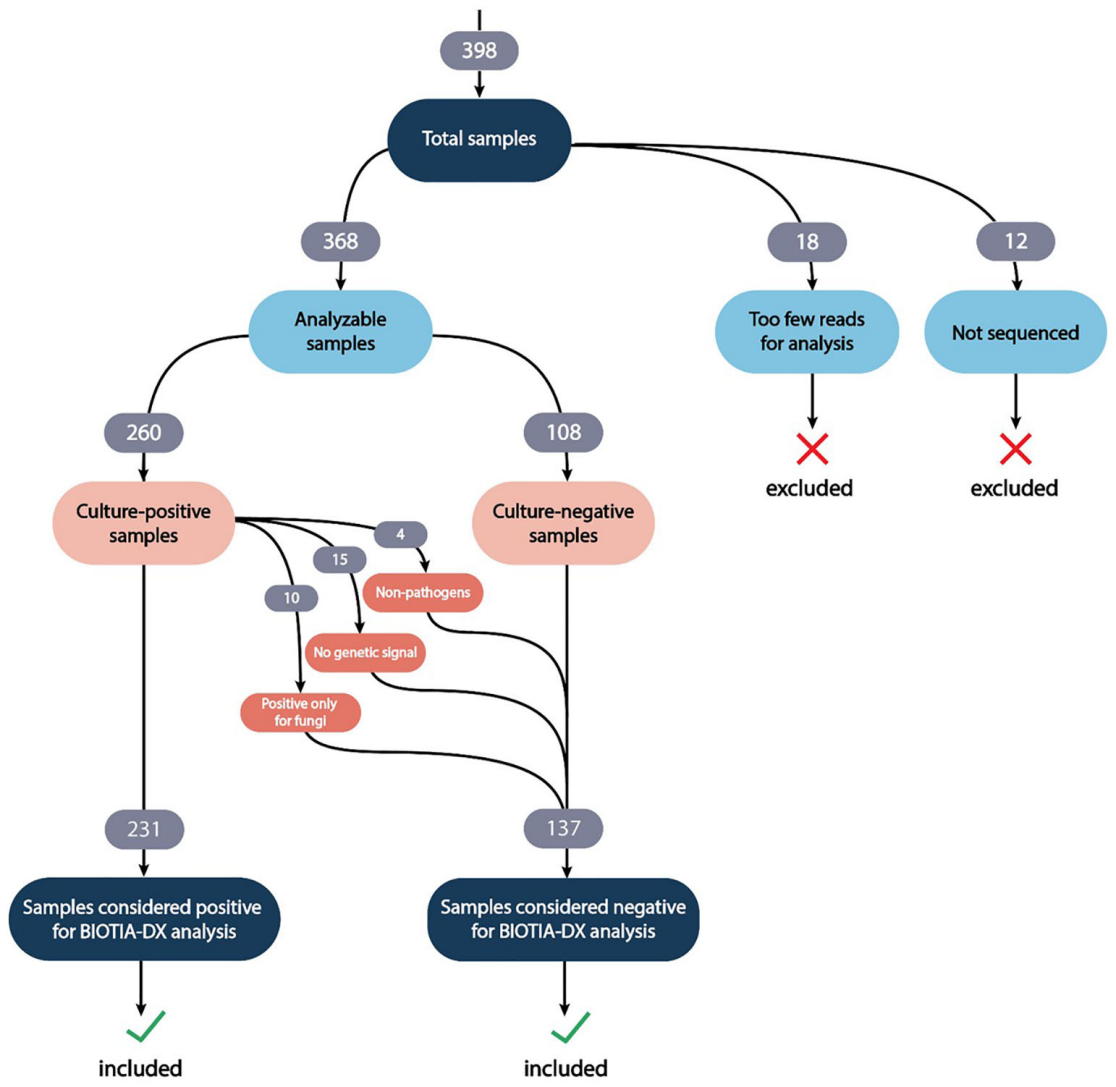
Study enrollment, sample processing workflow, and analytical framework.

Additionally, due to limitations in the extraction method, we were unable to analyze fungal targets using BIOTIA-DX; therefore, 10 samples positive only for a *Candida* species were also considered culture-negative. The remaining 2 samples with *Candida* identifications were culture-positive for an additional bacterial species. Finally, 4 organisms identified by culture are not considered typical causative agents of UTIs and were also excluded from BIOTIA-DX analysis (*Corynebacterium imitans* and *Lactobacillus* species). These re-classifications resulted in 231 culture-positive samples and 137 culture-negative samples (**Figure 1**).

We next then compared microbes identified by BIOTIA-DX to those identified by urine culture at both the sample and organism level. A complete list of all BIOTIA-DX identifications along with prediction probabilities is available in **Supplementary Table S2A**.

**Figure 2:**
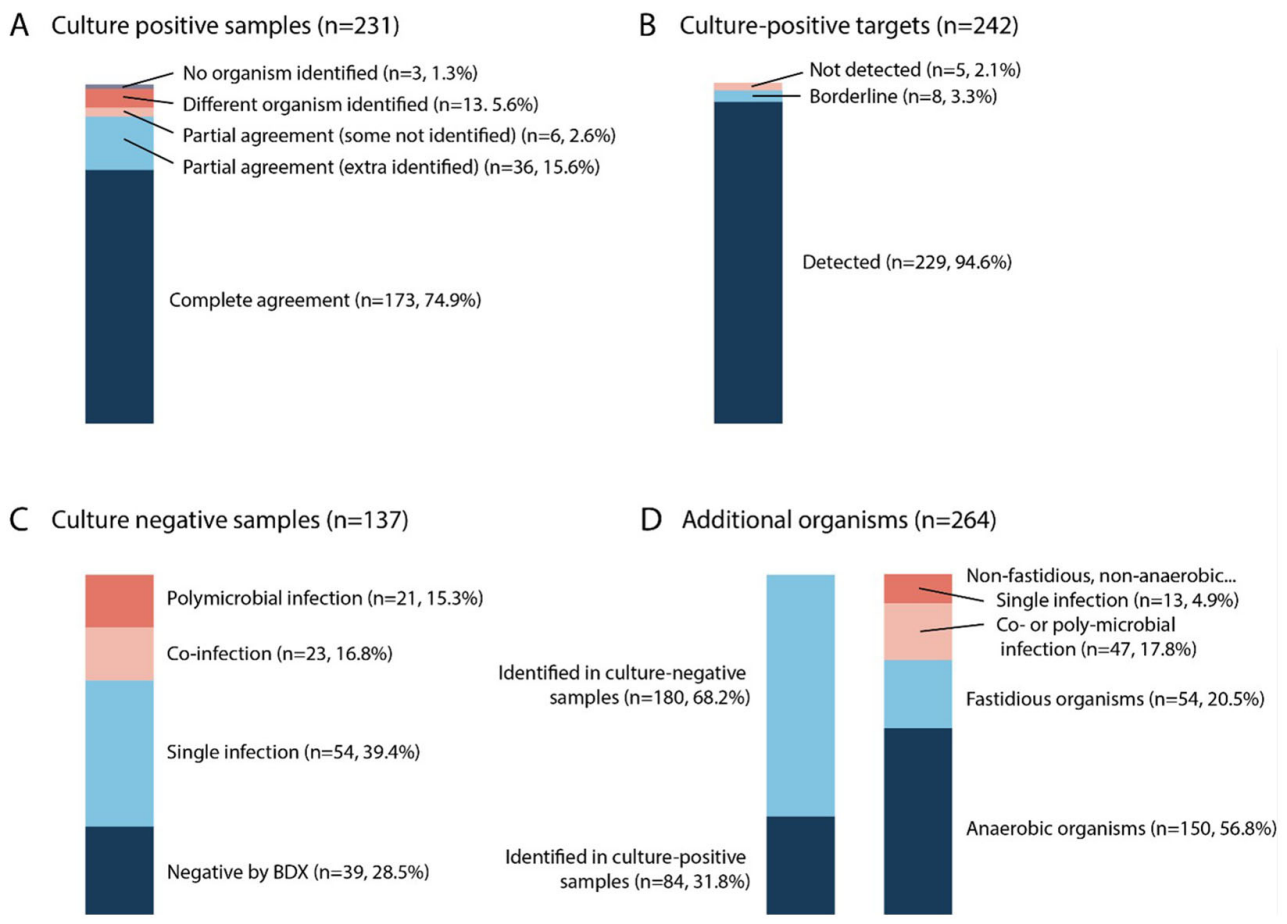
Fraction of samples and targets identified by culture and by BIOTIA-DX. **(A)** Fraction of culture-positive samples in complete, partial, or no agreement with BIOTIA-DX. **(B)** Fraction of culture-positive targets identified by BIOTIA-DX. **(C)** Fraction of culture-negative samples in which one or more organisms were identified by BIOTIA-DX. **(D)** Fraction of additional organisms identified by BIOTIA-DX found in culture-positive and -negative samples (left) and characteristics of additional organisms (right).

At the sample level, the comparison revealed a high degree of concordance for culture-positive samples. Of the 231 culture-positive samples, BIOTIA-DX identified at least one microbe in 228 samples (98.7%) and identified no microbes in 3 of the samples (1.3%) (**Figure 2A**). In samples where microbes were identified, 173 samples (74.9%) were in complete agreement between culture and BIOTIA-DX. In 36 cases (15.6%), BIOTIA-DX identified the culture-positive organism but also found additional organisms not identified by culture. In 13 cases (5.6%), at least one microbe identified by BIOTIA-DX was different than the one identified by culture. Finally, in 6 cases, BIOTIA-DX did not identify one of the organisms present in a co- or polymicrobial infection that was identified by culture (2.6%).

At the organism level, a total of 242 organisms were identified by urine culture across the 231 culture-positive samples (**Figure 2B**). BIOTIA-DX correctly identified 229 of these targets with confidence scores > 0.5, resulting in an overall organism-level sensitivity of 94.6% (**Supplementary Table S2A**). Notably, 5 of these targets were identified as a closely related species in the same genus by BIOTIA-DX (**Table 3**). Because of the likelihood that these were simply misclassified by culture, we considered these identifications to be true positives and in agreement with the culture result.

**Table 3:** Closely related species identified by culture vs. BIOTIA-DX.

Culture identified organism	BIOTIA-DX identified organism	Genus agreement	Notes
*Acinetobacter baumannii*	*Acinetobacter pittii*	Yes	Both in *A. baumannii* complex; difficult to differentiate phenotypically
*Escherichia coli*	*Escherichia fergusonii*	Yes	Closely related species; *E. fergusonii* shares >98% genetic similarity with *E. coli*
*Klebsiella pneumoniae*	*Klebsiella quasipneumoniae*	Yes	Both in *K. pneumoniae* complex; recently split taxonomically
*Staphylococcus aureus*	*Staphylococcus haemolyticus*	Yes	Different species; coagulase testing may explain discordance
*Staphylococcus aureus*	*Staphylococcus argentus*	Yes	*S. argenteus* recently split from *S. aureus* complex (2015)

There were 13 targets identified by culture but not by BIOTIA-DX due to prediction probability scores < 0.5 (**Table 4**). Of these 13 targets, more than half (n=8) had borderline scores between 0.4 and 0.5. Capturing these borderline calls would have increased the organism-level sensitivity from 94.6% to 97.9%. As BIOTIA-DX is trained on short-read sequencing data, these results indicate that the BDX assay is also highly effective at detecting the same pathogens identified by standard urine culture using long-read sequencing, which has a higher error rate than short-read technologies.

**Table 4:** Summary of organisms identified by culture but not identified by BIOTIA-DX. “Borderline” calls are organisms identified by the coarse classification step of BIOTIA-DX but with prediction probabilities between 0.4 - 0.5 in the fine classification step. The fourth column counts only cultured samples which passed inclusion criteria.

Organism	Not identified by BDX (score<0.4)	Borderline calls (score 0.4-0.5)	Total missed	Total cultured
*Escherichia coli*	3	3	6	154
*Enterococcus faecalis*	0	2	2	29
*Klebsiella pneumoniae*	0	1	1	23
*Citrobacter koseri*	0	1	1	4
*Acinetobacter baumannii*	1	0	1	3
*Staphylococcus epidermidis*	1	0	1	3
*Pseudomonas aeruginosa*	0	1	1	5

For culture-negative samples (n=137), a much greater degree of discordance was observed between culture and BIOTIA-DX. Only 39 of 137 (28.5%) culture-negative samples were found to be completely negative by BIOTIA-DX (**Supplementary Table S2A**). In the remaining 98 culture-negative samples, BIOTIA-DX identified one urogenital pathogen in 54 samples (39.4%), two co-infecting pathogens in 23 samples (16.8%), and polymicrobial infections of 3 or more pathogens in 21 samples (15.3%) (**Figure 2C**), demonstrating that mNGS identifies microbial signatures in a substantial number of cases deemed negative by the current standard. However, the clinical significance of these detections (infection vs. colonization vs. contamination) requires prospective validation with clinical correlation.

In total, across all 368 samples, BIOTIA-DX identified 264 organisms that were not identified by standard urine culture. The frequencies of organisms identified by BIOTIA-DX that were not identified by culture are shown in the second column of **Supplementary Table S2**. Eighty-four of the 264 additional identifications were identified in culture-positive samples known to contain at least one other organism, and 180 organisms were found across the 137 culture-negative samples. In general, the organisms identified by BIOTIA-DX that were not identified by standard urine culture were either anaerobic or fastidious organisms, with 204 out of the 264 detections (77.3%) falling into one of these two categories (**Figure 2D**).

The most commonly identified pathogen found by BIOTA-DX but not by culture was *Gardnerella vaginalis* (n=56, 15.2% of all samples) (**Table 5**; **Supplementary Table S2**), an anaerobic organism often associated with bacterial vaginosis (BV) that also plays a significant role in UTIs. Other anaerobic taxa that can cause UTIs frequently identified by BIOTIA-DX but not by culture included *Fannyhessea vaginae* (n=22, 6.0%), *Peptoniphilus* species (n=19, 5.2%), and *Anaerococcus* species (n=24, 6.6%). In total, 150 anaerobic organisms were identified in 80 different samples (21.7% of all samples, 56.8% of the 264 additional BIOTIA-DX identifications). While it is unclear which of these organisms may represent opportunistic infection or synergistic growth alongside true causative anaerobic organisms, anaerobic organisms nonetheless constitute a large burden of undiagnosed infections in UTI samples. Anaerobic urinary tract infections can be clinically serious, especially in the setting of complicated UTIs or immunocompromised patients, and are frequently missed by standard urine culture. No anaerobic organisms were detected by standard urine culture in the entire cohort, demonstrating a major diagnostic advantage of BIOTIA-DX over culture.

**Table 5:** Most commonly identified organisms by culture and/or BIOTIA-DX (Top 20 of 63 species).

Rank	Organism	Total positive by BDX and culture (% of all samples, N = 368)	Total positive by BDX only (% of total positive)	Anaerobic	Fastidious
1	*Escherichia coli*	133 (36.1%)	5 (3.8%)	No	No
2	*Gardnerella vaginalis*	56 (15.2%)	56 (100%)	Yes	–
3	*Enterococcus faecalis*	46 (12.5%)	19 (41.3%)	No	No
4	*Streptococcus anginosus*	24 (6.5%)	24 (100%)	No	Yes
5	*Fannyhessea vaginae*	22 (6.0%)	22 (100%)	Yes	-
6	*Klebsiella pneumoniae*	22 (6.0%)	1 (4.5%)	No	No
7	*Staphylococcus haemolyticus*	16 (4.3%)	13 (81.3%)	No	No
8	*Peptoniphilus harei*	13 (3.5%)	13 (100%)	Yes	–
9	*Streptococcus agalactiae*	11 (3.0%)	5 (45.5%)	No	Yes
10	*Staphylococcus epidermidis*	8 (2.2%)	6 (75.0%)	No	No
11	*Anaerococcus murdochii*	7 (1.9%)	7 (100%)	Yes	-
12	*Anaerococcus tetradius*	6 (1.6%)	6 (100%)	Yes	–
13	*Oligella urethralis*	6 (1.6%)	6 (100%)	No	Yes
14	*Proteus mirabilis*	6 (1.6%)	1 (16.7%)	No	No
15	*Enterobacter cloacae*	5 (1.4%)	0 (0%)	No	No
16	*Fusobacterium nucleatum*	5 (1.4%)	5 (100%)	Yes	–
17	*Streptococcus mitis*	5 (1.4%)	5 (100%)	No	Yes
18	*Acinetobacter baumannii*	4 (1.1%)	2 (50.0%)	No	No
19	*Actinotignum schaalii*	4 (1.1%)	4 (100%)	No	Yes
20	*Anaerococcus lactolyticus*	4 (1.1%)	4 (100%)	Yes	–
Subtotal (Top 20)		403 (109.5%)	207 (51.4%)		
Other organisms (n=43)		86 (23.4%)	57 (66.3%)		
TOTAL (all 63 species)		489 (132.9%)	264 (54.0%)		

The second most commonly identified pathogen found by BIOTIA-DX, but not by culture, was *Streptococcus anginosus* (n=24, 6.6% of all samples) (**Table 5**; **Supplementary Table S2**). *Streptococcus* species are facultatively anaerobic and considered to be fastidious, making them susceptible to missed detection via standard urine culture. Only *S. agalactiae* was identified by culture in 6 samples, and BIOTIA-DX identified it in an additional 5 samples, demonstrating it was potentially missed by culture in nearly 50% of cases. Following *S. anginosus* and *S. agalactiae*, viridans group streptococci were the third most commonly identified *Streptococcus* species (n=7, 1.9% of all samples), including *S. mitis*, *S. cristatus*, *S. parasanguinis*, and *S. oralis*. In total, BIOTIA-DX identified 40 cases (10.9% of all samples) of *Streptococcus* species that were not detected by culture. While *Streptococcus* species were the most common fastidious organism identified by BIOTIA-DX, other fastidious species identified by BIOTIA-DX included *Actinotignum* species (n=6), *Oligella urethralis* (n=6), and *Winkia neuii* (n=2). In total, 54 non-anaerobic fastidious organisms were identified (20.5% of the 264 additional BIOTIA-DX identifications). Together, these results demonstrate the additional significant advantage of BIOTIA-DX over urine culture in the identification of non-anaerobic fastidious organisms.

While anaerobic and fastidious organisms constituted the majority of BIOTIA-DX organisms not identified by culture, 60 of the 264 additional BIOTIA-DX detections were non-anaerobic and non-fastidious organisms identified across 51 unique samples (**Supplementary Table S2A**). For 47 of these 60 organisms (17.8% of the 264 additional BIOTIA-DX identifications), the infection was a co- or polymicrobial infection (n=38 samples). In 21 samples, a BIOTIA-DX organism was identified in addition to at least one co-infecting organism also identified by culture, and in 17 samples, none of the co- or polymicrobial infection organisms identified by BIOTIA-DX were detected by culture. In only 13 samples (3.5% of all samples, 4.9% of the 264 additional BIOTIA-DX identifications) was a single, non-anaerobic and non-fastidious organism identified by BIOTIA-DX but not by culture. In total, 284 detections of non-anaerobic non-fastidious organisms were made by either culture or BIOTIA-DX in this cohort, and with 60 identifications made only by BIOTIA-DX and not by culture, potentially 21.1% of all such infections may have been missed by culture. These results demonstrate that, even for non-anaerobic and non-fastidious organisms, BIOTIA-DX still has substantial utility over standard urine culture, particularly in the identification of co- and poly-microbial infections. The significance of these findings is amplified in hospitalized, catheterized, or immunocompromised patients, where co-infections are clinically meaningful and support more precise, risk-stratified treatment decisions.

The estimated sample-to-result timeline for the BDX platform is 11–13 hours. It supports the potential for same-day clinical reporting (< 24 hours), compared to the 24–48 hours typically required for standard urine culture. This includes a median of 5 hours for wet-lab processing (extraction and library preparation) followed by 6–8 hours of sequencing and automated analysis.

### 3.3 Performance of antimicrobial resistance prediction

#### 3.3.1 Urine AST results

Fluoroquinolone (178/287, 62.0%), beta-lactam (135/285, 47.4%), and sulfonamide/trimethoprim (105/239, 43.9%) resistance were the most frequently observed drug resistance types among the isolates. Aminoglycoside resistance occurred less frequently (26/274, 9.5%) and was primarily associated with *Enterococcus faecalis*. Data for tetracycline resistance via AST was not available. Among the fungal isolates, only *Candida krusei* demonstrated resistance to any of the tested antifungals (fluconazole). Rates of resistance identified by AST and BIOTIA-DX are shown in **Table 6**.

**Table 6:** Resistance rates observed for beta-lactam, sulfonamide/trimethoprim, aminoglycoside, fluoroquinolone, and tetracycline drugs.

Drug class	Resistance rate (all AST)	Resistance rate (inclusion criteria only)	BIOTIA-DX agreement	Frequency in culture-negative samples
Beta-lactam	135/285 (47.4%)	116/250 (46.4%)	106/116 (91.4%)	51/137 (37.2%)
Sulfonamide/trimethoprim	105/239 (43.9%)	92/209 (44.0%)	75/92 (81.5%)	4/137 (2.9%)
Aminoglycoside	26/274 (9.5%)	23/237 (9.7%)	21/23 (91.3%)	54/137 (39.4%)
Fluoroquinolone *(*E. Coli* only)	178/287 (62.0%)	102/128 (79.7%)	96/102 (94.1%)	-
Tetracycline	–	–	177/- (- %)	98/137 (71.5%)

Column 2 shows the overall resistance rate for all isolates tested via AST while column 3 shows only isolates which passed the inclusion criteria for BIOTIA-DX analysis. For fluoroquinolone analysis, only *E. coli* was able to be analyzed by BIOTIA-DX. Column 4 shows the number and frequency of isolates for which at least one ARG to that drug class was detected by BIOTIA-DX. Column 5 shows the number and frequency of isolates with resistance to the drug class in the culture-negative samples where a concurrent organism identification was also made by BIOTIA-DX. As only one isolate was phenotypically tested for tetracycline resistance, only the count of isolates for which a tetracycline ARG is reported in column 4.

#### 3.3.2 Beta-lactam resistance

In total, 285 isolates were tested for beta-lactam resistance; 135 (47.4%) were found to have an intermediate or resistant phenotype to at least one beta-lactam drug (cephalosporins, carbapenems, or penicillin). Of these, 250 passed the inclusion criteria for analysis by BIOTIA-DX. Not all isolates were tested for all drug classes. Of the samples suitable for BIOTIA-DX analysis, 116/250 (46.4%) showed a resistant or intermediate phenotype to at least one beta-lactam drug. BIOTIA-DX identified at least one beta-lactam ARG in 106/116 (91.4%) resistant isolates.

**Figure 3:**
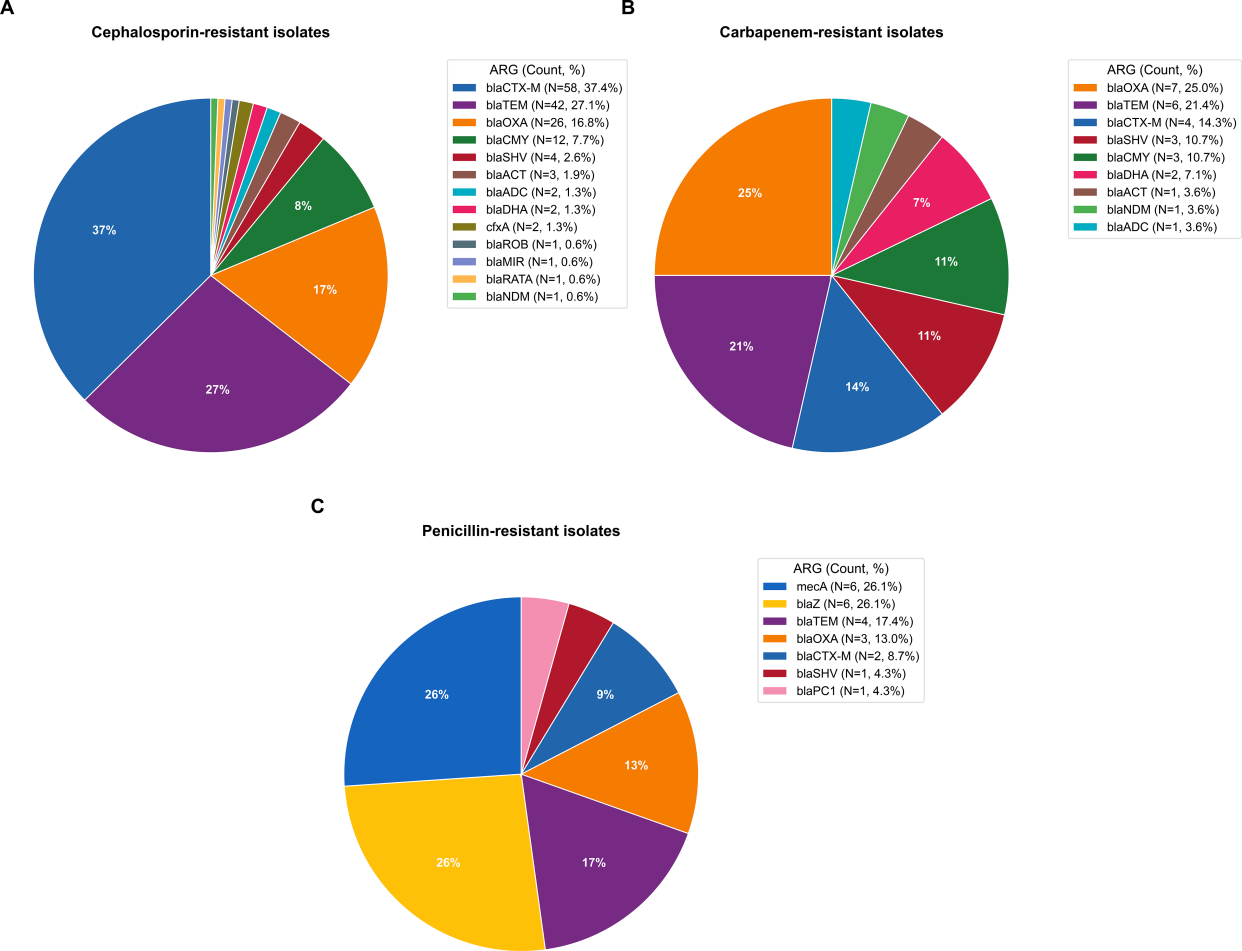
Beta-lactamase antimicrobial resistance gene profiles in phenotypically resistant bacterial isolates. **(A)** observed beta-lactamase ARGs in cephalosporin-resistant isolates. **(B)** observed beta-lactamase ARGs in carbapenem-resistant isolates. **(C)** observed ARGs in penicillin-resistant isolates.

Resistance against cephalosporins was the most common, with 78/246 (37.0%) of tested bacterial isolates showing a resistant or intermediate phenotype to at least one drug in this category (cefepime, ceftazidime, ceftriaxone, cefotaxime, or ceftaroline). The vast majority of cephalosporin resistance was observed in *E. coli*, where the resistance rate within the species exceeded 40% (72/166), and nearly 90% (64/72) of these carbapenem-resistant isolates exhibited extended-spectrum beta-lactamase (ESBL) activity. *Klebsiella pneumoniae*, another important UTI pathogen, exhibited very similar ARG patterns to *E. coli*, predominantly showing the presence of *bla*CTX-M, *bla*TEM, *bla*OXA, *bla*SHV, and *bla*CMY, but at a much lower phenotypic beta-lactam resistance rate (7/31). At least one beta-lactam resistance marker was detected by BIOTIA-DX in all but two (208/210, 99.0%) cephalosporin-resistant isolates, one *Pseudomonas aeruginosa* and one *Acinetobacter baumannii*, both of which were also false negative taxa detections by BIOTIA-DX. Observed beta-lactamase ARGs in cephalosporin-resistant isolates (**Figure 3A**) included *bla*CTX-M (n=58), *bla*TEM (n=42), *bla*OXA (n=26), *bla*CMY (n=12), *bla*SHV (n=4), blaACT (n=3), blaADC (n=2), blaDHA (n=2), *cfxA* (n=2), blaROB (n=1), blaMIR (n=1), blaRATA (n=1), and blaNDM (n=1).

Carbapenem resistance was the second most frequently observed beta-lactam resistance type observed in this cohort, with nearly 10% (22/233) of tested isolates showing a resistant or intermediate phenotype to a carbapenem drug (biapenem, meropenem, doripenem, imipenem, or ertapenem). Of the 233 isolates tested by AST, 198 passed inclusion criteria and were suitable for analysis by BIOTIA-DX. For 14 of the 19 (73.7%) carbapenem-resistant isolates that passed inclusion criteria, at least one beta-lactamase ARG was detected by BIOTIA-DX. Two of the five samples where no beta-lactam ARG was found but resistance was observed were the same *Pseudomonas aeruginosa* and *Acinetobacter baumannii* isolates as above. The remaining 3 discrepancies were two *Proteus mirabilis* isolates and one *Burkholderia cepacia*, two of which demonstrated only an intermediate level of resistance by AST. Beta-lactamase ARGs are observed in carbapenem-resistant isolates (**Figure 3B**) include *bla*OXA (n=7), *bla*TEM (n=6), *bla*CTX-M (n=4), *bla*SHV (n=3), *bla*CMY (n=3), *bla*DHA (n=2), *bla*ACT (n=1), *bla*NDM (n=1) and *bla*ADC (n=1). Among carbapenem-resistant *E. coli* strains, one was positive for *bla*OXA, *bla*CTX-M, and *bla*DHA, whereas the other (CRE phenotype) tested positive for *bla*TEM, *bla*CMY, and *bla*NDM. Both strains were also cephalosporin resistant.

Overall, 13 of 55 (23.6%) tested isolates showed a resistant phenotype to penicillin drugs (benzylpenicillin, ampicillin, oxacillin, or penicillin), and 52 were analyzable by BIOTIA-DX. Of these, 7 were various *Staphylococcus* species: *S. aureus*, *S. epidermidis*, *S. saprophyticus*, and *S. haemolyticus*. In all but two cases (50/52, 96.2%) were no beta-lactamase gene detected by BIOTIA-DX in a penicillin-resistant isolate (one *S. saprophyticus* and one *Enterococcus faecium*). Observed ARGs in penicillin-resistant isolates (**Figure 3C**) include *mecA* (n=6), *bla*Z (n=6), *bla*TEM (n=4), *bla*OXA (n=3), *bla*CTX-M (n=2), *bla*SHV (n=1), and *bla*PC1 (n=1).

Notably, *bla*EC, a chromosomally encoded class C beta-lactamase in *E. coli*, was detected in more than 90% of *E. coli* isolates regardless of phenotype. Excluding *bla*EC, BIOTIA-DX found 76/265 (28.7%) instances where an isolate was susceptible to all tested beta-lactam drugs but was positive for at least one beta-lactam ARG. In 18 of 76 (23.7%) cases, these additional ARG identifications may have been due to the presence of at least one co-infecting organism present in the clinical sample. In the remaining 58 cases (76.3%), it is possible the isolate was simply not tested for the beta-lactam drug to which it may have been resistant. In total, only 10/250 isolates tested resistant for at least one beta-lactam drug, but no ARGs were detected by BIOTIA-DX, three of which were also false negative taxa detections, and two of which only demonstrated an intermediate level of drug resistance.

#### 3.3.3 Trimethoprim/sulfamethoxazole and aminoglycoside resistance

In total, 239 isolates were tested via AST for combination sulfamethoxazole/trimethoprim, and 209 of these passed inclusion criteria for analysis by BIOTIA-DX. Of these, 92/209 (44.0%) were resistant to sulfamethoxazole/trimethoprim by AST. Resistance is typically caused by horizontal gene transfer and acquisition of *sul* (sulfonamide resistance) and *dfr* (trimethoprim resistance) genes, which are often present on plasmids. As these drugs were only tested in combination, we required both genes to be detected by BIOTIA-DX for a sample to be considered resistant. At least one *sul* and *dfr* gene was detected in 75/92 (81.5%) resistant isolates. In 12 of 92 (13.0%) cases either *sul* or *dfr* were identified, but not both, and in only 5 cases was neither gene identified in a resistant isolate. Three of the cases where BIOTIA-DX did not identify both genes were also false negative taxa calls by BIOTIA-DX. In susceptible isolates, the pipeline-AST agreement was 89% (57/62), with 5 samples showing detection of both *sul* and *dfr* genes despite susceptible phenotypes.

In total, 274 isolates were tested for aminoglycoside resistance by AST, 26 (9.5%) of which were resistant to at least one aminoglycoside drug (amikacin, gentamicin, or streptomycin). The highest level of aminoglycoside resistance was observed in *Enterococcus faecalis* (18/26, 69.2%). In total, 237 samples passed inclusion criteria for analysis by BIOTIA-DX and 23 (9.7%) showed an intermediate or resistant phenotype. Four plasmid-borne markers frequently reported in resistant *Enterococcus* species were detected: *aph*(3’)-IIIa aminoglycoside phosphotransferase (16/18), *aac*(6’)-Ie aminoglycoside acetyltransferase (14/18), *ant*(9’)-Ia aminoglycoside nucleotidyltransferase (4/18), and *ant*(6’)-Ia aminoglycoside nucleotidyltransferase (16/19). In all but two cases (21/23, 91.3%) at least one aminoglycoside ARG was detected by BIOTIA-DX for all resistant or intermediate isolates (both *E. faecalis*). There were no cases where BIOTIA-DX detected aminoglycoside ARGs in isolates that tested susceptible to all aminoglycoside drugs.

#### 3.3.4 Fluoroquinolone resistance in *E. coli* isolates

Currently, the BIOTIA-DX pipeline supports fluoroquinolone resistance detection only in *E. coli*. The pipeline reports common mutations in *gyrA*, *gyrB*, *parC*, and *parE* proteins that confer resistance to fluoroquinolones. Additionally, a machine learning classifier predicts resistance phenotype based on the mutation distributions in these proteins, and plasmid-mediated quinolone resistance markers are also reported. BIOTIA-DX only predicts susceptible or resistant phenotypes and does not include an intermediate category.

Of the 287 isolates tested for quinolone resistance, 128 were *E. coli* isolates that passed inclusion criteria for analysis by BIOTIA-DX. Based on the phenotypic AST, the resistance rate among *E. coli* isolates was 79.7% (102/128), with 13 (10.2%) samples classified as intermediate and 26 (20.3%) as susceptible. Among the susceptible isolates, the machine learning model correctly predicted all 26 cases. For resistant isolates, 86/89 (96.6%) were correctly predicted as resistant by BIOTIA-DX. For intermediate isolates, 3 were predicted as susceptible and 10 as resistant, resulting in an overall agreement for resistant or intermediate phenotypes of 96/102 (94.1%). Two common quinolone resistance-determining regions (QRDR) mutations in *gyrA* (S83L, D87N) and one in *parC* (S80I) were dominant in resistant isolates (**Table 7**). Additionally, *qnrS*, a plasmid-mediated quinolone resistance protein, was detected in 6 isolates. Among the intermediate isolates (n=13), the *gyrA* S83L mutation was the most frequent (n=8), and the *qnr* marker was also present (n=6).

**Table 7:** Fluoroquinolone resistance-associated mutations in *E. coli* isolates (N = 128).

Mutation	Frequency in resistant (n=89)	Frequency in susceptible (n=26)	Frequency in intermediate (n=13)
gyrA (S83L)	81 (91%)	6 (23%)	8 (61%)
parC (S80I)	83 (93%)	0 (0%)	0 (0%)
gyrA (D87N)	78 (87%)	0 (0%)	0 (0%)
parE (I529L)	33 (37%)	2 (7%)	1 (7%)
parC (E84V)	31 (35%)	0 (0%)	0 (0%)
parE (L416F)	28 (31%)	0 (0%)	0 (0%)
parE (S458A)	13 (14%)	0 (0%)	0 (0%)

#### 3.3.5 Tetracycline resistance

Only one isolate was tested for tetracycline resistance, so we were unable to compare the performance of BIOTIA-DX to AST for tetracycline. Overall, BIOTIA-DX identified at least one tetracycline ARG in 177 culture-positive samples. The most frequently observed markers included tet(M) (n=96), tet(A) (n=78), tet(L) (n=27), tet(B) (n=27), tet(C) (n=18), tet(O) (n=17), tet(K) (n=14), mepA (n=8), tet(D) (n=8), tet(J) (n=7), tet(W) (n=7), and tet(Q) (n=6).

### 3.4 ARG profiles in culture-negative samples

A total of 137 samples were considered to be culture-negative for BIOTIA-DX analysis. Within these samples, BIOTIA-DX predicted the presence of at least one ARG in 96 samples. Beta-lactam ARGs were found in 63/137 (46.0%) culture-negative samples, with 51 (37.2%) occurring in samples where BIOTIA-DX made at least one positive taxa call. Among beta-lactam ARGs, *blaZ* (n=27), *mecA* (n=18), and their operon elements were commonly detected. In 11/18 detections of mecA, a *Staphylococcus* species was also identified as additional taxa call by BIOTIA-DX. Other markers including *bla*TEM (n=32), *cfxA* (n=8), *bla*CTX-M (n=3), *bla*SHV (n=1), and *bla*OXA (n=3) were also identified. Either *sul* (n=12) or *dfr* (n=17) were detected in 24 (17.5%) culture-negative samples, 19 (13.9%) of which were also taxa-positive by BIOTIA-DX. Of these, 5 showed the presence of both *sul* and *dfr* genes, four of which were also taxa-positive. Aminoglycoside ARGs were detected in 73 (53.3%) culture-negative samples, 54 (39.4%) of which were taxa-positive by BIOTIA-DX. For aminoglycoside markers, *aph*(3’)-Ia (n=42), *aph*(3’)-IIIa (n=29), and *ant*(6)-Ia (n=28) were the most prevalent ARGs identified. Tetracycline resistance markers were the most prevalent in the culture-negative sample set (n=98, 71.5%) most commonly including *tet*(M) (n=80), *tet*(L) (n=34), and *tet*(W) (n=26).

## 4 Discussion

This study represents the first analytical validation of the BIOTIA-DX metagenomic platform (mNGS) in a Southeast Asian population. Our findings demonstrate its successful transportability and robust performance outside of its original development cohort. The platform achieved high sensitivity, with 98.7% of culture-positive samples found to be positive by BIOTIA-DX and 94.6% of culture organisms correctly identified. Its performance is on par with other leading mNGS platforms in the field (Almas et al., 2023), indicating that its underlying databases and analytical models are broadly applicable and can reliably detect common uropathogens in a non-US patient population, showing strong concordance with culture-based methods. This provides the first and most critical evidence for the platform’s global applicability and supports its use in diverse clinical settings worldwide.

Furthermore, while BIOTIA-DX is commercially established on Illumina sequencing platforms in a CLIA lab, this study represents the first validation of its use with Oxford Nanopore technology, demonstrating its adaptability to a platform that offers near real-time analysis capabilities. Because of the higher error rate of Nanopore sequencing, the high sensitivity achieved by BIOTIA-DX in this study is notable, considering it was trained on short-read sequencing data. Given that 8 of the false negative calls identified by culture but missed by BIOTIA-DX were borderline with confidence scores between 0.4-0.5, additional development and training of the machine learning algorithm with Nanopore data could improve the sensitivity from 94.6% to 97.9% by capturing these borderline identifications.

The most significant finding regarding the platform’s clinical utility is its ability to identify microbial DNA in samples that were negative by urine culture, with 98 out of 137 culture-negative samples (71.5%) showing evidence of microbial infection. In particular, BIOTIA-DX excelled at identifying anaerobic and fastidious organisms which are often negative by culture. In total, BIOTIA-DX made 264 microbial identifications that were not found by culture, and 204 (77.2%) of these were either anaerobic or fastidious. This capability is particularly crucial for guiding therapy in symptomatic, culture-negative patients, a common and challenging clinical scenario.

The 7.5% (n=30) sample exclusion rate may be attributed to a combination of low microbial biomass in urine samples exacerbated by specific wet-lab processing steps. DNA was extracted promptly upon sample receipt and subsequently stored at -20 °C to facilitate batching for library preparation. This delay may have induced minor DNA fragmentation, particularly on low microbial loads or those from patients receiving antibiotic therapy. Furthermore, fragmented or low-abundance microbial DNA libraries or host DNA dominance can result in insufficient microbial sequencing depth. This may explain why 22 of the 30 excluded samples maintained acceptable total DNA concentrations yet failed to reach the 500 non-human read threshold. However, it is important to note that these samples were processed in 2019–2020 using a protocol that has since been continued to be refined. The 2025 wet-lab protocol has been significantly improved to reduce the failure rate and to improve the detection of fungal organisms. Thus, further validation using optimized sample processing methods is recommended to reduce the sample failure rate in future studies.

The most commonly identified organism found by BIOTIA-DX in culture-negative samples was *Gardnerella vaginalis*, found in over 15% of all samples in this cohort. *G. vaginalis* is an anaerobic organism frequently associated with both recurrent UTI and bacterial vaginosis (BV) potentially serving as a causative agent in culture-negative symptomatic patients. BV is typically characterized by a decrease in beneficial *Lactobacillus* species in the vaginal microbiome and an overgrowth of anaerobic organisms, causing a dysbiosis that leads to symptomatic presentation. Many of these organisms can also infect the urinary tract and cause UTIs, often associated with recurrent UTIs. In addition to *G. vaginalis*, BIOTIA-DX also identified 24 other species of anaerobic, BV-associated organisms, including *Fannyhessea vaginae*, *Anaerococcus* species, and *Peptoniphilus* species. In total, anaerobic organisms were present in 80 samples, 21.7% of all samples in this cohort. Under-diagnosis of anaerobic organisms in UTIs is a critical gap in diagnostic accuracy and clinical care, particularly for women’s health.

BIOTIA-DX also excelled at identifying fastidious organisms, particularly *Streptococcus* species, that were not identified by urine culture. In total, 40 identifications of *Streptococcus* were made by BIOTIA-DX (10.9% of all samples), only 6 of which were identified by culture. In addition to *Streptococcus* species, 5 other fastidious species were identified in this cohort, including *Actinotignum* species, *Oligella urethralis*, and *Winkia neuii*, resulting in a total of 54 detections of fastidious organisms.

Further, where BIOTIA-DX made identifications of organisms that were non-anaerobic and non-fastidious, more than 60% of these identifications were made in co- or polymicrobial infections. The identification of polymicrobial infections, in particular, may explain cases where treatment targeted at a single, dominant pathogen fails, highlighting the need for a more comprehensive diagnostic approach. Even in samples that were positive by urine culture, BIOTIA-DX identified additional organisms that may represent co- or polymicrobial infections in 36 out of 231 samples (15.6%). Approximately 25% of all non-anaerobic and non-fastidious organisms identified in this cohort were found only by BIOTIA-DX and may have potentially been missed by urine culture, demonstrating that BIOTIA-DX has significant utility even for non-anaerobic and non-fastidious organisms.

We also found five cases where culture and BIOTIA-DX agreed on the genus of an organism but disagreed on the exact species. Because phenotypic differentiation between closely related species is probably less reliable than genotypic differentiation, we suspect that the BIOTIA-DX call identified the correct species which was misidentified by culture. We considered these calls to be true positives and in agreement between the two methods because species within these complexes share proteomic profiles that frequently default to the most clinically prevalent taxa during MALDI-TOF identification. Without a third independent test to establish a consensus ground truth, we utilized this classification to reflect the analytical concordance at the genus level while noting the refined resolution provided by the genotypic approach. These cases demonstrate the utility of BIOTIA-DX over culture in differentiating between closely related species.

While our study successfully identifies a hidden burden of anaerobic and fastidious organisms, a future challenge will be to establish clear clinical guidelines for when and how to treat these non-traditional uropathogens in the context of a UTI. Further research correlating these findings with patient outcomes is needed to translate these powerful diagnostic results into clear therapeutic pathways.

The platform’s AMR prediction capabilities provide actionable insights, particularly in a high-burden setting like Thailand especially on first-line antibiotics. For the drug classes tested, BIOTIA-DX achieved high accuracy at identifying ARGs in samples that were phenotypically intermediate or resistant by AST. BIOTIA-DX was most accurate in detecting fluoroquinolone resistance in *E. coli* (94%) and least effective at identifying resistance to sulfamethoxazole/trimethoprim combination treatment (81.5%). Detection of beta-lactam and aminoglycoside resistance were both ~91%.

The metagenomic data provides the local antimicrobial resistance landscape within this cohort. The striking prevalence of tetracycline resistance genes, particularly tet(M) and tet(A), is a key finding, as tetracycline susceptibility is not routinely tested in many standard urinalysis panels, potentially masking a widespread resistance reservoir. For beta-lactams, the dominance of the *bla*CTX-M gene confirms a high burden of extended-spectrum beta-lactamase (ESBL) producers, which directly explains the high rates of cephalosporin resistance observed phenotypically. Similarly, the frequent detection of the gyrA (S83L) mutation provides a clear genetic correlation for the rampant fluoroquinolone resistance endemic to the region. The detection of *bla*NDM carbapenemase genes in this cohort demonstrates the high prevalence of carbapenem resistance and highlights BIOTIA-DX’s ability to identify clinically significant, high-risk resistance genes. The detailed view of the local “resistome” demonstrates the dual utility of mNGS: not only as a diagnostic for individual patients but also as a powerful surveillance tool to inform and refine regional antimicrobial stewardship guidelines.

It is important to note that the detection of a gene associated with resistance to a drug class does not always correlate with phenotypic resistance to all drugs in that drug class. The functional activity of these genes can vary, and not all genes are active against all drugs in a drug class. For example, not all beta-lactamase genes will be effective against carbapenems, but may be effective against other beta-lactam drugs such as cephalosporins. This highlights the need for careful interpretation of genomic resistance data alongside published phenotypic susceptibility for specific genes in order to make the correct interpretation in a clinical context (Sawa et al., 2020).

For samples that were susceptible by AST, it is more difficult to assess the congruence between the methods due to the fact that not all isolates were subjected to AST for all drugs. Therefore, an isolate that was found to be susceptible to all drugs tested but where ARGs were identified may not have been tested for the drug to which the ARG confers resistance. Additionally, it is not possible to connect ARGs to specific organisms in the sample, so the ARGs identified by BIOTIA-DX for samples with isolates that were susceptible to all tested drugs may belong to other organisms in the sample. While assessing AMR via AST requires testing each individual drug, assessing AMR via the presence of ARGs can be done in a single test.

Notably, the platform’s ability to cover challenging pathogens like *Pseudomonas aeruginosa*, a ‘superbug’ of increasing clinical concern, further warrants its future robustness and utility in addressing the next wave of AMR challenges. The successful application in this new population suggests that the platform’s core technology is generalizable.

Adapting the BIOTIA-DX platform to Oxford Nanopore technology provides a significant reduction in sample-to-result turnaround time (TAT). Based on established protocol benchmarks, a total TAT of 11–13 hours is achievable, which compares favorably to the standard 24–48 hours required for conventional urine culture. This emphasizes the platform’s potential to support same-day clinical decision-making, particularly in urgent or complicated UTI cases where rapid intervention is critical.

## 5 Limitations

First, the proprietary, “black box” nature of the BIOTIA-DX pipeline presents a challenge for academic transparency and reproducibility. While our results support its external validity, the inability to interrogate the specific algorithms or training data of the machine learning models remains a constraint.

Second, like all DNA-based sequencing methods, our approach detects microbial genetic material but cannot distinguish between viable, infecting pathogens and DNA from colonizing, commensal, or dead organisms (Simner et al., 2018; Gu et al., 2019). Therefore, a positive mNGS result requires careful correlation with clinical signs of infection. In particular, BIOTIA-DX may be identifying genetic material from commensal skin bacteria, such as *Staphylococcus* species. However, there were a total of 23 organisms detected by culture that had no significant genetic material detected by BIOTIA-DX at all. Typically, when culture organisms are missed by BIOTIA-DX, they are detected in the coarse classification step but fail to exceed a prediction probability > 0.5 during the machine learning fine classification step. It is exceedingly rare for a true positive organism to fail to pass coarse classification, which supports that the culture identification may have been a false positive by culture. This demonstrates that, while NGS-based approaches are commonly criticized as producing too many false positive identifications, the gold-standard culture method is not itself immune from false positives, either (Bermudez et al., 2025; He et al., 2024).Third, with the lack of a third diagnostic method such as PCR to establish a consensus ground truth, we cannot be certain whether the additional calls made by BIOTIA-DX were true positives missed by culture or false positives incorrectly called by the algorithm. Likewise, we were unable to determine if culture-positive samples with no detected genomic signal were false positives by culture or true positive samples that failed extraction or sequencing steps. Additionally, we were unable to measure specificity or positive predictive value of the BIOTIA-DX test for this study and could only calculate the sensitivity of culture-identified organisms. Future prospective validation studies should incorporate pre-specified orthogonal testing protocols to establish a definitive consensus ground truth.

Fourth, in polymicrobial infections, the metagenomic approach identifies a pooled profile of AMR genes and cannot definitively link a specific resistance gene to a specific pathogen in the sample. This presents a challenge for targeted therapy in co-infections.

Fifth, this study focused on the analytical performance of the assay and did not measure the total turnaround time. While this study provides an estimated protocol benchmark of 11–13 hours for the BDX workflow, we did not formally measure the real-world clinical turnaround time (from sample collection to physician notification) for each retrospective case in this cohort. Future implementation studies should assess the complete sample-to-result time to determine its utility in guiding initial empirical antibiotic therapy, although <24h is possible.

Sixth, The BIOTIA-DX output is a qualitative result based on a machine learning confidence score, and unlike urine culture, it does not provide a quantitative measure of microbial load (CFU/mL) to differentiate colonization from infection.

Seventh, the accuracy of any sequencing-based test relies on the comprehensiveness of its reference databases. While the platform performed well, the potential for geographic bias in public and proprietary databases, which may under-represent strains or AMR variants unique to Southeast Asia, remains a consideration.

Another point to note is that the 7.5% sample exclusion rate due to insufficient non-human reads represents a limitation inherent to mNGS testing of clinical samples with variable microbial loads. This rate is consistent with published literature for clinical mNGS platforms and reflects the fundamental challenge of detecting microbial DNA against a high human DNA background (Janes et al., 2022; Francisco et al., 2023). Strategies to mitigate these challenges have been optimized within the wet-lab protocol since the time this study was conducted, thereby reducing the overall failure rate and enabling the inclusion of fungal organisms in future studies.

Finally, this study was conducted in a single private hospital, and its findings may not be fully generalizable to public hospitals or other regions within Thailand. In addition, the study was retrospective and did not include any interventions that would enable analysis of whether diagnosis with BIOTIA-DX leads to improved treatment and improved patient outcomes compared to diagnosis by urine culture.

## 6 Conclusions

Despite its limitations, this study provides strong evidence for the successful international deployment of an advanced diagnostic platform. This analytical validation study demonstrates that the BIOTIA-DX metagenomic sequencing platform achieves robust concordance with standard urine culture in a Thai tertiary hospital population. The platform demonstrated high sensitivity at both the sample level (98.7%) and organism level (94.6%), indicating that its underlying databases and analytical models are broadly applicable beyond the original US development cohort. This represents the first validation of BIOTIA-DX using Oxford Nanopore Technology and the first validation in a Southeast Asian population, providing evidence for the platform’s transportability across geographic regions and sequencing technologies. This work is a critical step towards globalizing the fight against AMR through advanced, validated diagnostic technologies.

## Data Availability

The original contributions presented in the study are included in the article/**Supplementary Material**. Further inquiries can be directed to the corresponding authors.
