# Correction: Analytical validation of a metagenomic next-generation diagnostic platform for urinary tract infection in a Thai tertiary hospital setting: a BI-Biotia UTI cohort study

**DOI:** 10.3389/fcimb.2026.1817909

**Published:** 2026-03-17

**Authors:** Panupong Wangprapa, Dorottya Nagy-Szakal, Heather L. Wells, Gabor Fidler, Montinee Sangtian, Wipa Panmontha, Srichan Bunlungsup, Wichai Techasathit, Mara Couto-Rodriguez, David C. Danko, Christopher E. Mason, Niamh B. O’Hara, Sira Sriswasdi, Teeradache Viangteeravat

**Affiliations:** 1 Bumrungrad International Hospital, Bangkok, Thailand; 2 Inter-Department Program of Biomedical Sciences, Faculty of Graduate School, Chulalongkorn University, Bangkok, Thailand; 3 Center of Excellence in Computational Molecular Biology, Faculty of Medicine, Chulalongkorn University, Bangkok, Thailand; 4 Genomic Medicine Center, Bumrungrad International Hospital, Bangkok, Thailand; 5 Biotia Inc., New York, NY, United States; 6 The Department of Cell Biology, College of Medicine, SUNY Downstate Health Sciences University, New York, NY, United States; 7 VitalLife Scientific Wellness Center, Bangkok, Thailand; 8 Center for Artificial Intelligence in Medicine, Research Affairs, Faculty of Medicine, Chulalongkorn University, Bangkok, Thailand

**Keywords:** antibiotic stewardship, antimicrobial resistance, high risk UTI patients, long read sequencing, metagenomic sequencing, Oxford Nanopore Technology, urinary tract infection

Affiliation Biotia Inc., New York, NY, United States was omitted for author Mara Couto-Rodriguez. This affiliation has now been added for author Mara Couto-Rodriguez.

Affiliation Biotia Inc., New York, NY, United States was omitted for author David C. Danko. This affiliation has now been added for author David C. Danko.

Affiliation Biotia Inc., New York, NY, United States was omitted for author Christopher E. Mason. This affiliation has now been added for author Christopher E. Mason.

Affiliation Biotia Inc., New York, NY, United States was omitted for author Niamh B. O’Hara This affiliation has now been added for author Niamh B. O’Hara.

Author Mara Couto-Rodriguez was erroneously assigned to affiliation Center of Excellence in Computational Molecular Biology, Faculty of Medicine, Chulalongkorn University, Bangkok, Thailand.

This affiliation has now been removed for author Mara Couto-Rodriguez.

Author David C. Danko was erroneously assigned to affiliation Center of Excellence in Computational Molecular Biology, Faculty of Medicine, Chulalongkorn University, Bangkok, Thailand.

This affiliation has now been removed for author David C. Danko.

Author Christopher E. Mason was erroneously assigned to affiliation Center of Excellence in Computational Molecular Biology, Faculty of Medicine, Chulalongkorn University, Bangkok, Thailand.

This affiliation has now been removed for author Christopher E. Mason.

Author Niamh B. O’Hara was erroneously assigned to affiliation Center of Excellence in Computational Molecular Biology, Faculty of Medicine, Chulalongkorn University, Bangkok, Thailand.

This affiliation has now been removed for author Niamh B. O’Hara.

