# Supplementary material for: Analytical validation of a metagenomic next-generation diagnostic platform for urinary tract infection in a Thai tertiary hospital setting: a BI-Biotia UTI cohort study
Source: Front Cell Infect Microbiol. 2026 Feb 13;16:1751074. doi: 10.3389/fcimb.2026.1751074 (PMC12947133; doi:10.3389/fcimb.2026.1751074)
Supplement: Supplementary file 1 [file Table1.pdf]

Supplementary Table S1

Cohort characteristics with a detailed nationality breakdown (N = 398)

Characteristic	Overall Cohort (N = 398)
**Age**	
Mean (SD)	62.53 (18.38)
Median (IQR)	64.0 (49.2–76.0)
**Age Group, n (%)**	
<20 years	0 (0.0%)
20–29 years	15 (3.8%)
30–39 years	41 (10.3%)
40–49 years	44 (11.1%)
50–59 years	64 (16.1%)
60–69 years	77 (19.3%)
70–79 years	78 (19.6%)
80–89 years	58 (14.6%)
≥90 years	21 (5.3%)
**Sex, n (%)**	
Female	277 (69.6%)
Male	121 (30.4%)
**Nationality, n (%)**	
Thailand	161 (40.7%)
Myanmar	51 (12.9%)
United Arab Emirates	18 (4.5%)
Oman	18 (4.5%)
Bangladesh	18 (4.5%)
Qatar	16 (4.0%)
United States of America	13 (3.3%)
United Kingdom	11 (2.8%)
Cambodia	10 (2.5%)
Kuwait	10 (2.5%)
Japan	8 (2.0%)
China	7 (1.8%)
Australia	4 (1.0%)
Ethiopia	4 (1.0%)
Vietnam	3 (0.8%)
Unknown	5 (1.3%)
Germany	3 (0.8%)
Romania	2 (0.5%)
Egypt	2 (0.5%)
Saudi Arabia	2 (0.5%)
Nepal	2 (0.5%)
Pakistan	2 (0.5%)
Canada	2 (0.5%)
Sweden	2 (0.5%)
Switzerland	2 (0.5%)
India	2 (0.5%)
Sri Lanka	1 (0.3%)
Ireland	1 (0.3%)
Netherlands	1 (0.3%)
Korea (South)	1 (0.3%)
Israel	1 (0.3%)

Characteristic	Overall Cohort (N = 398)
Singapore	1 (0.3%)
France	1 (0.3%)
Poland	1 (0.3%)
Indonesia	1 (0.3%)
Austria	1 (0.3%)
Maldives	1 (0.3%)
Yemen	1 (0.3%)
Italy	1 (0.3%)
Jordan	1 (0.3%)
Chad	1 (0.3%)
Lao	1 (0.3%)
Russian Federation	1 (0.3%)
Estonia	1 (0.3%)
Malaysia	1 (0.3%)
Mongolia	1 (0.3%)

SD, standard deviation; IQR, interquartile range. Nationality data based on patient registration information.
