# Supplementary material for: Analytical validation of a metagenomic next-generation diagnostic platform for urinary tract infection in a Thai tertiary hospital setting: a BI-Biotia UTI cohort study
Source: Front Cell Infect Microbiol. 2026 Feb 13;16:1751074. doi: 10.3389/fcimb.2026.1751074 (PMC12947133; doi:10.3389/fcimb.2026.1751074)
Supplement: Supplementary file 2 [file Table2.pdf]

Supplementary Table S2

Summary of all organisms identified by culture or by BIOTIA-DX (BDX). Counts include only organisms that passed inclusion criteria. The first column is the number of samples positive for each organism by either culture or BIOTIA-DX and the percentage is this value divided by the total number of samples in the cohort (N=368). The second column is the number of positive samples that were positive by BIOTIA-DX only, and the percentage is this value divided by the total number of samples positive from the first column.

Organism	Total positive by BDX and culture (% of all samples)	Total positive by BDX only (% of total positive)	Anaerobic	Fastidious
Acinetobacter baumannii	4 (1.09%)	2 (50%)	No	No
Acinetobacter pittii	1 (0.27%)	0 (0%)	No	No
Actinotignum sanguinis	1 (0.27%)	1 (100%)	No	Yes
Actinotignum schaalii	4 (1.10%)	4 (100%)	No	Yes
Actinotignum urinale	1 (0.27%)	1 (100%)	No	Yes
Anaerococcus hydrogenalis	1 (0.27%)	1 (100%)	Yes	-
Anaerococcus lactolyticus	4 (1.10%)	4 (100%)	Yes	-
Anaerococcus murdochii	7 (1.92%)	7 (100%)	Yes	-
Anaerococcus prevotii	3 (0.82%)	3 (100%)	Yes	-
Anaerococcus tetradius	6 (1.65%)	6 (100%)	Yes	-
Anaerococcus vaginalis	3 (0.82%)	3 (100%)	Yes	-
Atopobium deltae	1 (0.27%)	1 (100%)	Yes	-
Bacteroides fragilis	3 (0.82%)	3 (100%)	Yes	-
Burkholderia cepacia	1 (0.27%)	0 (0%)	No	No
Campylobacter ureolyticus	2 (0.55%)	2 (100%)	Yes	-
Citrobacter freundii	2 (0.55%)	1 (50%)	No	No
Citrobacter koseri	3 (0.82%)	0 (0%)	No	No
Eggerthella lenta	1 (0.27%)	1 (100%)	Yes	-
Enterobacter cloacae	5 (1.37%)	0 (0%)	No	No
Enterococcus faecalis	46 (12.50%)	19 (41.3%)	No	No
Enterococcus faecium	4 (1.10%)	0 (0%)	No	No
Enterococcus raffinosus	2 (0.55%)	1 (50%)	No	No
Escherichia coli	133 (36.14%)	5 (3.8%)	No	No
Escherichia fergusonii	2 (0.55%)	1 (50%)	No	No
Fannyhessea vaginae	22 (6.0%)	22 (100%)	Yes	-
Fusobacterium nucleatum	5 (1.37%)	5 (100%)	Yes	-
Gardnerella vaginalis	56 (15.22%)	56 (100%)	Yes	-
Hoylesella timonensis	3 (0.82%)	3 (100%)	Yes	-
Klebsiella aerogenes	2 (0.55%)	1 (50%)	No	No
Klebsiella pneumoniae	22 (6.0%)	1 (4.5%)	No	No
Klebsiella quasipneumoniae	3 (0.82%)	2 (66.6%)	No	No
Klebsiella variicola	2 (0.55%)	2 (100%)	No	No
Mobiluncus curtisii	4 (1.10%)	4 (100%)	Yes	-
Mobiluncus mulieris	1 (0.27%)	1 (100%)	Yes	-
Morganella morganii	3 (0.82%)	1 (33.3%)	No	No
Oligella urethralis	6 (1.65%)	6 (100%)	No	Yes
Peptoniphilus duerdenii	1 (0.27%)	1 (100%)	Yes	-
Peptoniphilus gorbachii	2 (0.55%)	2 (100%)	Yes	-
Peptoniphilus harei	13 (3.57%)	13 (100%)	Yes	-
Peptoniphilus lacrimalis	3 (0.82%)	3 (100%)	Yes	-
Prevotella bivia	2 (0.55%)	2 (100%)	Yes	-
Proteus mirabilis	6 (1.65%)	1 (16.7%)	No	No
Pseudomonas aeruginosa	4 (1.10%)	0 (0%)	No	No
Serratia marcescens	1 (0.27%)	0 (0%)	No	No
Sneathia sanguinegens	2 (0.55%)	2 (100%)	Yes	-
Staphylococcus argenteus	1 (0.27%)	0 (0%)	No	No
Staphylococcus aureus	4 (1.10%)	1 (25%)	No	No

Organism	Total positive by BDX and culture (% of all samples)	Total positive by BDX only (% of total positive)	Anaerobic	Fastidious
Staphylococcus epidermidis	8 (2.20%)	6 (75%)	No	No
Staphylococcus haemolyticus	16 (4.40%)	13 (81.3%)	No	No
Staphylococcus hominis	2 (0.55%)	2 (100%)	No	No
Staphylococcus lugdunensis	1 (0.27%)	1 (100%)	No	No
Staphylococcus saprophyticus	1 (0.27%)	0 (0%)	No	No
Streptococcus agalactiae	11 (3.02%)	5 (45.5%)	No	Yes
Streptococcus anginosus	24 (6.52%)	24 (100%)	No	Yes
Streptococcus cristatus	1 (0.27%)	1 (100%)	No	Yes
Streptococcus dysgalactiae	1 (0.27%)	1 (100%)	No	Yes
Streptococcus gallolyticus	2 (0.55%)	2 (100%)	No	Yes
Streptococcus mitis	5 (1.37%)	5 (100%)	No	Yes
Streptococcus parasanguinis	1 (0.27%)	1 (100%)	No	Yes
Streptococcus salivarius	1 (0.27%)	1 (100%)	No	Yes
Veillonella montpellierensis	2 (0.55%)	2 (100%)	Yes	-
Veillonella parvula	3 (0.82%)	3 (100%)	Yes	-
Winkia neuii	2 (0.55%)	2 (100%)	No	Yes
**TOTAL (63 species)**	**489**	**264 (54.0%)**		

BDX, BIOTIA-DX. Anaerobic: Yes indicates obligate anaerobic organism not routinely detected by standard aerobic culture. Fastidious: Yes indicates organism requiring specialized growth conditions. '-' indicates not applicable (anaerobic organisms are categorized separately from fastidious).
